# Supplementary material for: Integrating Systematic Surveys With Historical Data to Model the Distribution of Ornithodoros turicata americanus, a Vector of Epidemiological Concern in North America
Source: Ecol Evol. 2024 Nov 11;14(11):e70547. doi: 10.1002/ece3.70547 (PMC11554405; doi:10.1002/ece3.70547)
Supplement: Supplementary file 1 — Appendix S1. Appendix S2. Appendix S3. Appendix S4. [file ECE3-14-e70547-s001.docx]

**Supporting Information**

**S1: Environmental covariates**

**Bioclimatic variables dataset**

| **Abbreviation** | **Definition** |
| --- | --- |
| BIO1 | Annual Mean Temperature |
| BIO2 | Mean Diurnal Range (Mean of monthly (max temp - min temp)) |
| BIO3 | Isothermality (BIO2/BIO7) (×100) |
| BIO4 | Temperature Seasonality (standard deviation ×100) |
| BIO5 | Max Temperature of Warmest Month |
| BIO6 | Min Temperature of Coldest Month |
| BIO7 | Temperature Annual Range (BIO5-BIO6) |
| BIO8 | Mean Temperature of Wettest Quarter |
| BIO9 | Mean Temperature of Driest Quarter |
| BIO10 | Mean Temperature of Warmest Quarter |
| BIO11 | Mean Temperature of Coldest Quarter |
| BIO12 | Annual Precipitation |
| BIO13 | Precipitation of Wettest Month |
| BIO14 | Precipitation of Driest Month |
| BIO15 | Precipitation Seasonality (Coefficient of Variation) |
| BIO16 | Precipitation of Wettest Quarter |
| BIO17 | Precipitation of Driest Quarter |
| BIO18 | Precipitation of Warmest Quarter |
| BIO19 | Precipitation of Coldest Quarter |

**Table S1.1.** Bioclimatic variables from the BIOS+ dataset of the CHELSA V2.1 initiative (Brun et al., 2022)

**Figure S1.1.** Loadings of each variable for the first five principal components of the PCA summarizing the climatic condition within the study area.

**Soil conditions dataset**

| **Variable name** | **Description** | **Mapped units** | **Conversion factor** | **Conventional units** |
| --- | --- | --- | --- | --- |
| bdod | Bulk density of the fine earth fraction | cg/cm³ | 100 | kg/dm³ |
| cec | Cation Exchange Capacity of the soil | mmol(c)/kg | 10 | cmol(c)/kg |
| cfvo | Volumetric fraction of coarse fragments (> 2 mm) | cm3/dm3 (vol‰) | 10 | cm3/100cm3 (vol%) |
| clay | Proportion of clay particles (< 0.002 mm) in the fine earth fraction | g/kg | 10 | g/100g (%) |
| nitrogen | Total nitrogen (N) | cg/kg | 100 | g/kg |
| phh2o | Soil pH | pHx10 | 10 | pH |
| sand | Proportion of sand particles (> 0.05 mm) in the fine earth fraction | g/kg | 10 | g/100g (%) |
| silt | Proportion of silt particles (≥ 0.002 mm and ≤ 0.05 mm) in the fine earth fraction | g/kg | 10 | g/100g (%) |
| soc | Soil organic carbon content in the fine earth fraction | dg/kg | 10 | g/kg |
| ocd | Organic carbon density | hg/m³ | 10 | kg/m³ |
| ocs | Organic carbon stocks | t/ha | 10 | kg/m² |

**Table S1.2.** Variables describing soil properties from the SoilGrids Database (Poggio et al., 2021).

**Figure S1.2.** Loadings of each variable for the first two principal components of the PCA summarizing the physicochemical characteristics of the soil.


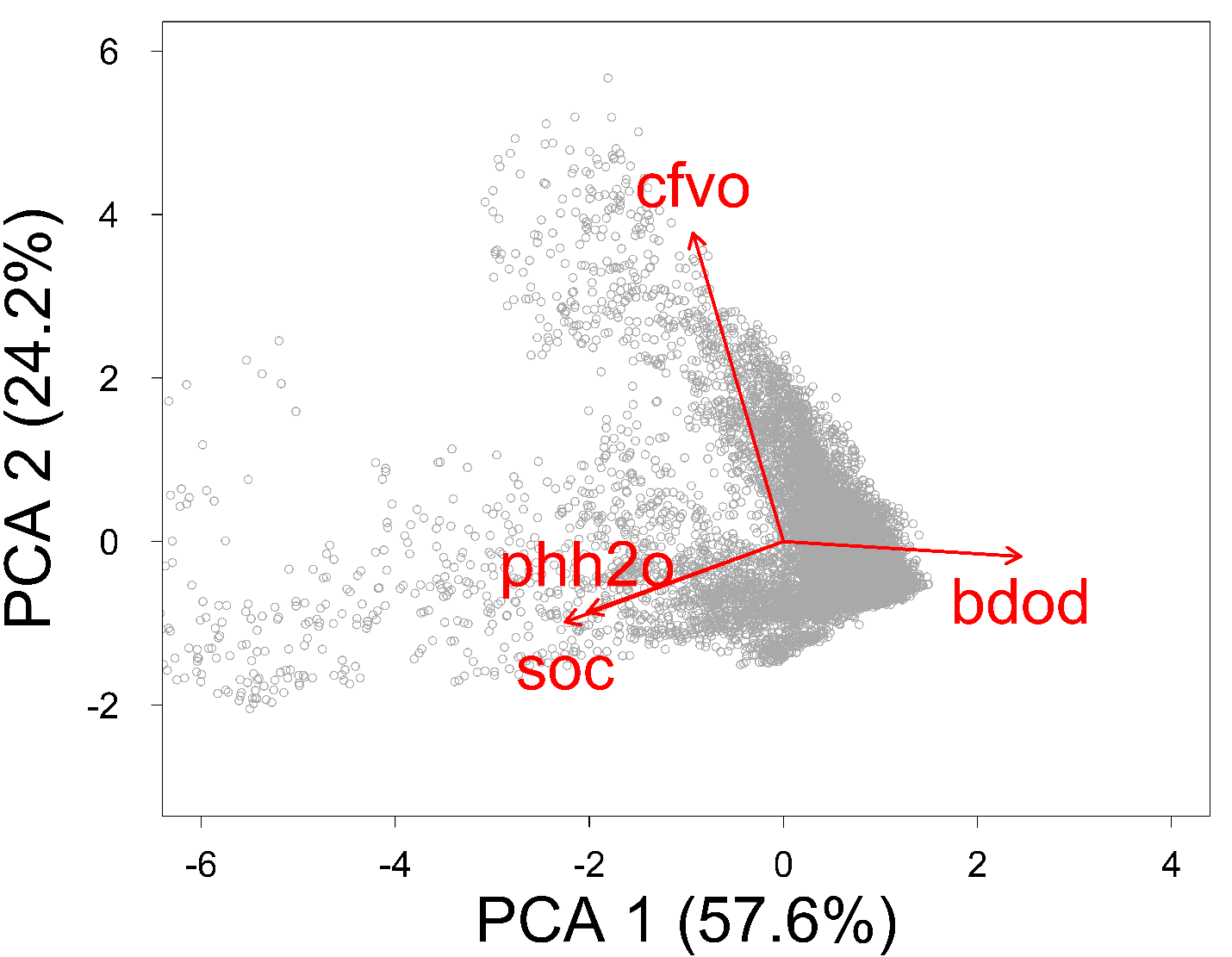


**Figure S1.3.** Biplot of the first two principal components of the PCA summarizing the physicochemical characteristics of the soil.


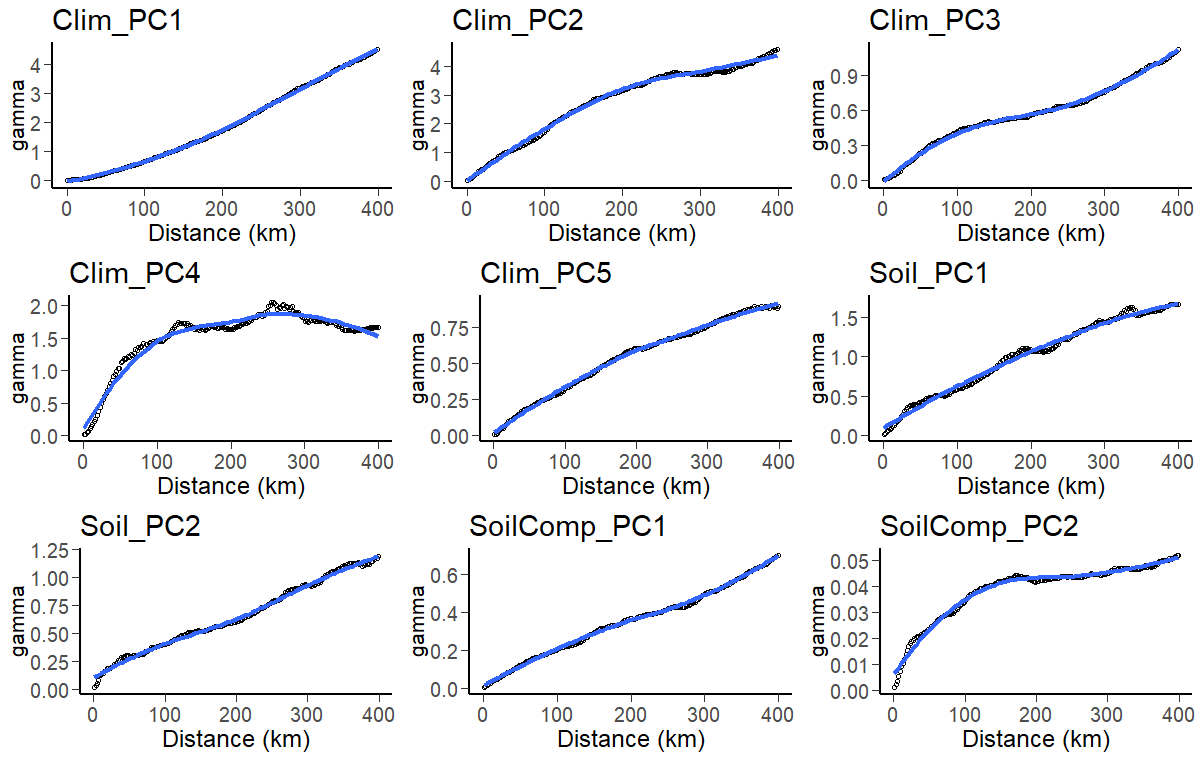


**Figure S1.4.** Spatial autocorrelation of each environmental variable. Variograms were created using a lag of 2 km and a maximum distance of 400 km. The open dots display the calculated gamma value at each interval, while the blue line shows a fitted local regression line. These variograms show spatial autocorrelation in all variables up to and above 100 km.

**S2: Sampling design**


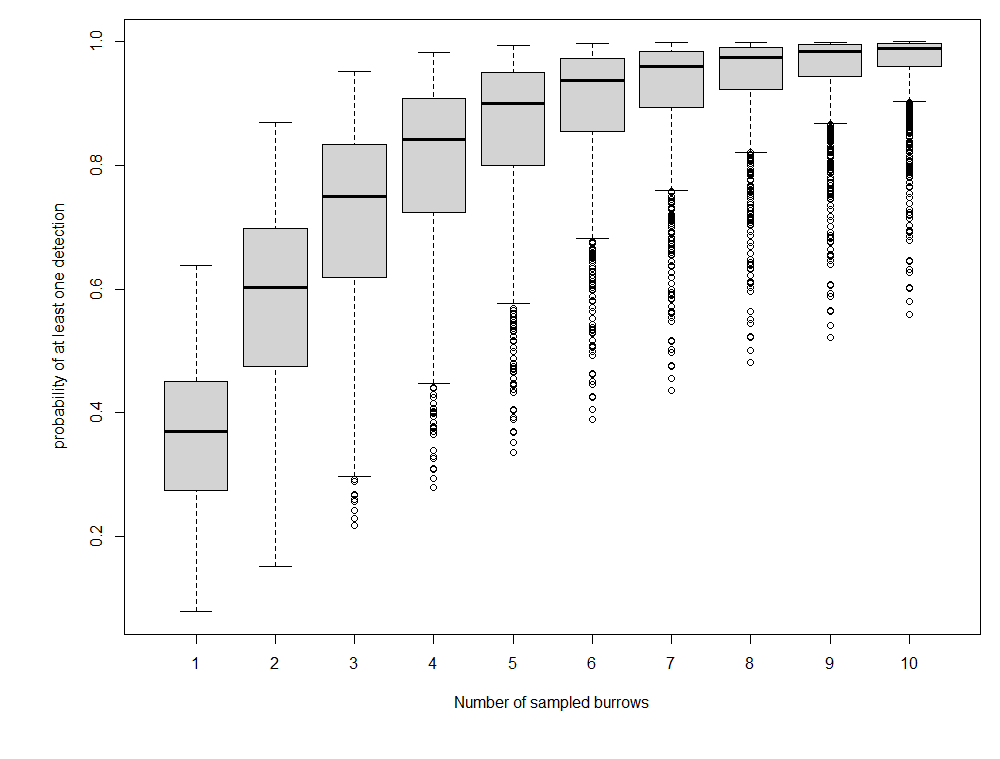


**Figure S2.** Probability of detecting *O. turicata* at a site as a function of the number of sampled burrows. The probability of detection was estimated using the local burrow occupancy and tick detection probabilities estimated by Canino et al. (2024).

**S3: Model evaluation**

**Figure S3.** Double checkboard pattern of occurrence data partitioning for model validation. Each color represents one partition that alone or in conjunction with another partition was used to evaluate the model create with the remaining data in ten replicates.

**S4: Maxent model variable importance and response**

| **Variable** | **Percent contribution** | **Permutation importance** |
| --- | --- | --- |
| SoilComp_PC1 | 51.3 | 88.7 |
| Clim_PC1 | 43 | 2.3 |
| Clim_PC5 | 2.4 | 2.5 |
| Clim_PC4 | 1.1 | 1 |
| Soil_PC1 | 1 | 2.5 |
| Clim_PC3 | 0.6 | 1.2 |
| Clim_PC2 | 0.4 | 1 |
| Soil_PC2 | 0.2 | 0.7 |

**Table S4.** Two metrics of variable importance estimated from the Maxent models. To determine the first estimate, in each iteration of the training algorithm, the increase in regularized gain is added to the contribution of the corresponding variable, or subtracted from it if the change to the absolute value of lambda is negative. For the second estimate, for each environmental variable in turn, the values of that variable on training presence and background data are randomly permuted. The model is reevaluated on the permuted data, and the resulting drop in training AUC is shown in the table, normalized to percentages.

**
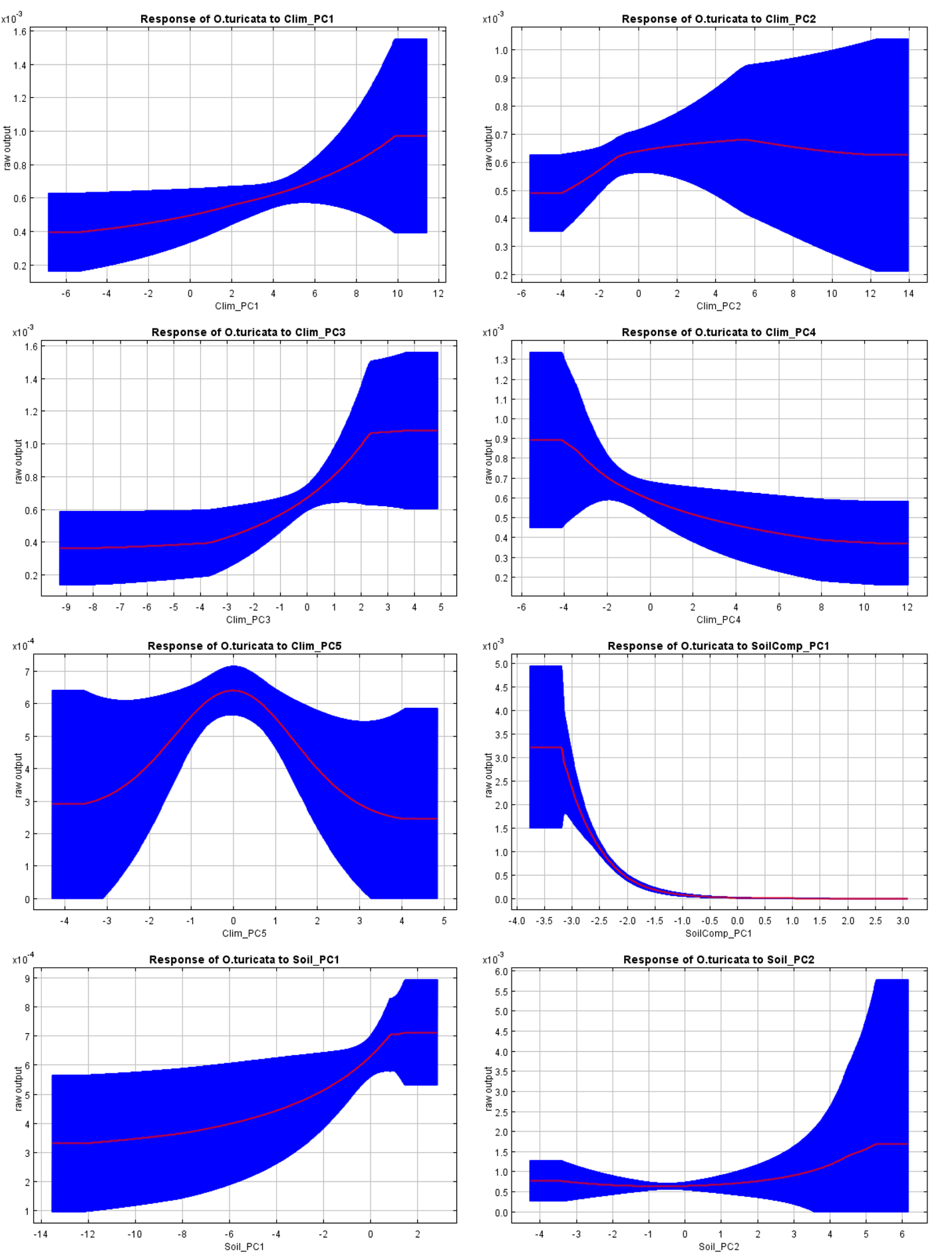
**

**Figure S4.** Marginal response curves from 50 Maxent model replicates representing how the predicted probability of presence changes as each environmental variable is varied, keeping all other environmental variables at their average sample value.

**References**

Brun, P., Zimmermann, N.E., Hari, C., Pellissier, L., Karger, D. 2022: Data from: CHELSA-BIOCLIM+ A novel set of global climate-related predictors at kilometre-resolution. **EnviDat.**<https://doi.org/10.16904/envidat.332>.

Canino, N., Torhorst, C., Botero‐Cañola, S., Beati, L., O'Hara, K.C., James, A. and Wisely, S.M. 2024. Development of a rapid and reliable surveillance method for *Ornithodoros turicata americanus* in gopher tortoise (*Gopherus polyphemus*) burrows in the southeastern United States. Medical and Veterinary Entomology, 2024 Sep 11. doi: 10.1111/mve.12764. Epub ahead of print. PMID: 39258964.

Poggio, L., de Sousa, L. M., Batjes, N. H., Heuvelink, G. B. M., Kempen, B., Ribeiro, E., and Rossiter, D. 2021. SoilGrids 2.0: producing soil information for the globe with quantified spatial uncertainty, SOIL, 7, 217–240, 2021.
